# Evaluation of hepatic functional reserve of hepatocellular carcinoma (≤ 5 cm) by liver shear wave velocity combined with multiple parameters

**DOI:** 10.3389/fonc.2024.1301052

**Published:** 2024-03-14

**Authors:** Long Yang, Haohui Zhu, Xijun Zhang, Xiaobing Fu, Xiaojuan Zhao, Xiaojing Wang, Xiaozhuan Ren, Haibo Yu, Jianjun Yuan

**Affiliations:** ^1^ Department of Ultrasound, Zhengzhou University People’s Hospital, Henan Provincial People’s Hospital, Zhengzhou, China; ^2^ Department of Hepatobiliary and Pancreatic Surgery, Zhengzhou University People’s Hospital, Henan Provincial People’s Hospital, Zhengzhou, China

**Keywords:** hepatocellular carcinoma, hepatic functional reserve, ultrasound, elastography, indocyanine green clearance test

## Abstract

**Background:**

Normal hepatic functional reserve is the key to avoiding liver failure after liver surgery. This study investigated the assessment of hepatic functional reserve using liver shear wave velocity (LSWV) combined with biochemical indicators, tumor volume, and portal vein diameter.

**Methods:**

In this single-center prospective study, a total of 123 patients with hepatocellular carcinoma (HCC) were divided into a test group (n=92) and a validation group (n=31). All patients were Child-Pugh grade A. The indocyanine green retention rate at 15 min (ICG-R15), liver shear wave velocity (LSWV), portal vein diameter (D_pv_), alanine aminotransferase (ALT), aspartate transaminase (AST), alkaline phosphatase (ALP), γ-glutamyl transpeptidase (γ-GGT), albumin (ALB), prothrombin time (PT), and also liver tumor volume (maximum diameter ≤5 cm) were measured. In the test group, multiple parameters were used to evaluate hepatic functional reserve, and the multiparametric model was established. Receiver operating characteristic (ROC) curve analysis was conducted to assess the diagnostic performance of the multiparametric model. In the validation group, the predictive effectiveness of the multiparametric model was analyzed using consistency tests.

**Results:**

It was revealed that LSWV, ALB, and PT were statistically significant in evaluation of the hepatic functional reserve (*P*<0.05). The multiparametric model was formulated as follows: Y= -18.954 + 9.726*LSWV-0.397*ALB+2.063*PT. The value of the area under the curve (AUC) for the multiparametric model was 0.913 (95% confidence interval (CI): 0.835-0.962, *P<* 0.01), with a cutoff value of 16.656 (sensitivity, 0.763; specificity, 0.926). The Kappa value of consistency testing was 0.655 (*P*<0.01).

**Conclusion:**

LSWV combined with ALB and PT exhibited a high predictive effectiveness for the assessment of hepatic functional reserve, assisting the clinical diagnosis and management of liver diseases.

## 1 Introduction

Currently, the hepatocellular carcinoma (HCC) cases in China account for 55% of the world’s HCC cases (1), and the incidence is the fourth highest among all malignant diseases in China (2). The high incidence of HCC in China is closely associated with the high incidence of hepatitis B virus (HBV) infection (3). Surgery is still the main therapeutic strategy for HCC patients (4). A poor hepatic functional reserve is prevalent among a number of HCC patients with HBV infection, which is an important cause of postoperative liver failure and perioperative death (5). Therefore, preoperative evaluation of hepatic functional reserve and appropriate management are critical for reducing perioperative mortality (6).

Hepatic functional reserve, which refers to the potential liver function in response to increased physiological stress on the liver, is the liver remnant functional capacity after liver injury. It mainly depends on the number of functional hepatocytes and the integrity of the structure of hepatic tissues (7). The quality of hepatic functional reserve reflects the strength of the liver’s compensatory capacity. At present, indocyanine green (ICG) clearance, score system, and liver volume measurement are common methods to assess liver function. Liver volume measurement is always more expensive than the other two methods. Wang YY’s study shows that ICG-R15 is more accurate than the Child-Pugh score and Model for End-Stage Liver Disease (MELD) score in liver functional reserve assessment (8). The ICG clearance test is a commonly used test in Asian countries (9, 10) and ICG retention rate at 15 min (ICG-R15) is the most common preoperative test for the evaluation of hepatic functional reserve (11). However, ICG-R15 is susceptible to portal venous blood flow, serum bilirubin levels, and other factors. The diagnostic accuracy of ICG-R15 may be impacted by portal vein thrombosis, portal vein cavernous degeneration, or abnormal bilirubin levels (12).

It has been confirmed that liver shear wave velocity (LSWV) and multiple clinical factors can assess preoperative hepatic functional reserve. While recent studies have concentrated on LSWV as a single index to assess hepatic functional reserve, other factors have been neglected (11, 13–15). The present study aimed to investigate the use of liver shear wave velocity (LSWV) combined with multiple factors to assess the hepatic functional reserve. Although ICG-R15 has some shortcomings, due to its widespread clinical application and its advantage over the other two methods, we chose it as the gold standard for hepatic reserve function in this study.

## 2 Materials and methods

### 2.1 Patients

**Figure 1:**
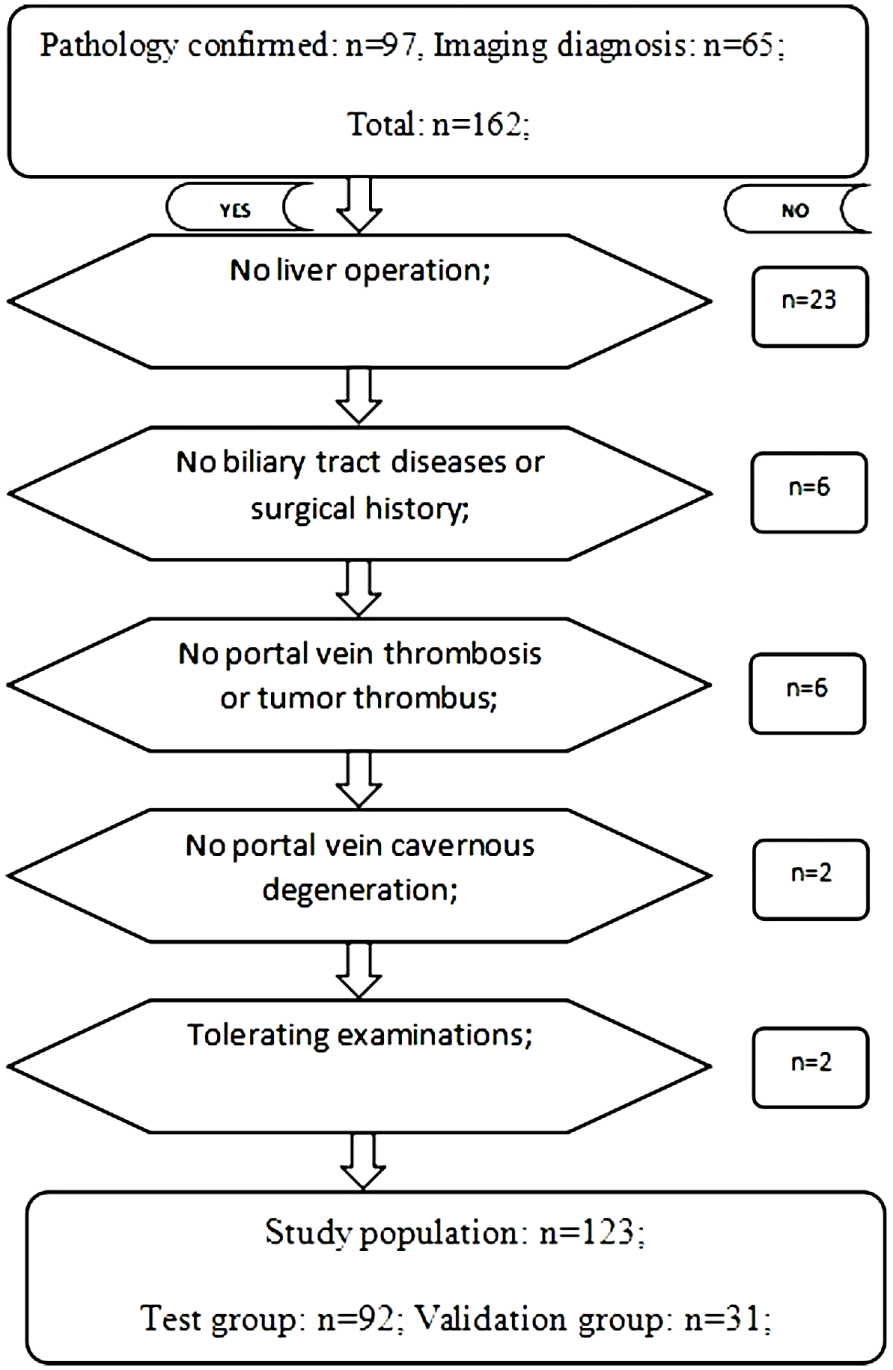
Flowchart of the study.

This single-center prospective study was approved by our Institutional Review Board (IRB) (IRB No. 58). From June 2018 to December 2021, 162 patients with HCC-complicated HBV infection admitted to Henan Provincial People’s Hospital met the inclusion criteria. All patients with hepatocellular carcinoma were diagnosed by biopsy pathology, surgery pathology, or imaging examination. Inclusion criteria: 1. Biopsy or surgery pathology confirmed hepatocellular carcinoma; 2. Imaging diagnosis of hepatocellular carcinoma; 3. Hepatitis B virus infection; 4. Patient has single or multiple hepatic nodules. The upper limit diameter of a single tumor was ≤ 5 cm, or the sum of the maximum diameter of multiple tumors was ≤ 5 cm; 5. Patient has Child-Pugh grade A; 6. Patient has a normal range of direct bilirubin, indirect bilirubin, and total bilirubin (12); 7. Patient’s liver enzymes (AST and/or ALT) are within five times of the upper normal limits (16); and 8. Patients are able to complete LSWV measurement and other related examinations. Exclusion criteria: 1. Hepatic surgical history; 2. Biliary tract diseases or surgical history; 3. Portal vein thrombosis or tumor thrombus; 4. Cavernous degeneration of portal vein; and 5. Liver congestion, acute hepatitis, and infiltrative liver disease (16). In total, 123 patients (male (99) vs. female (24), with an age range of 31-81 years old) were enrolled. By using the random number table method, patients were randomly divided into a test group (n=92) and a validation group (n=31) in a ratio of 3:1. The test group was used to build the model, and the validation group was used to assess the predictive effectiveness of the model (**Figure 1**).

### 2.2 LSWV examination

**Figure 2:**
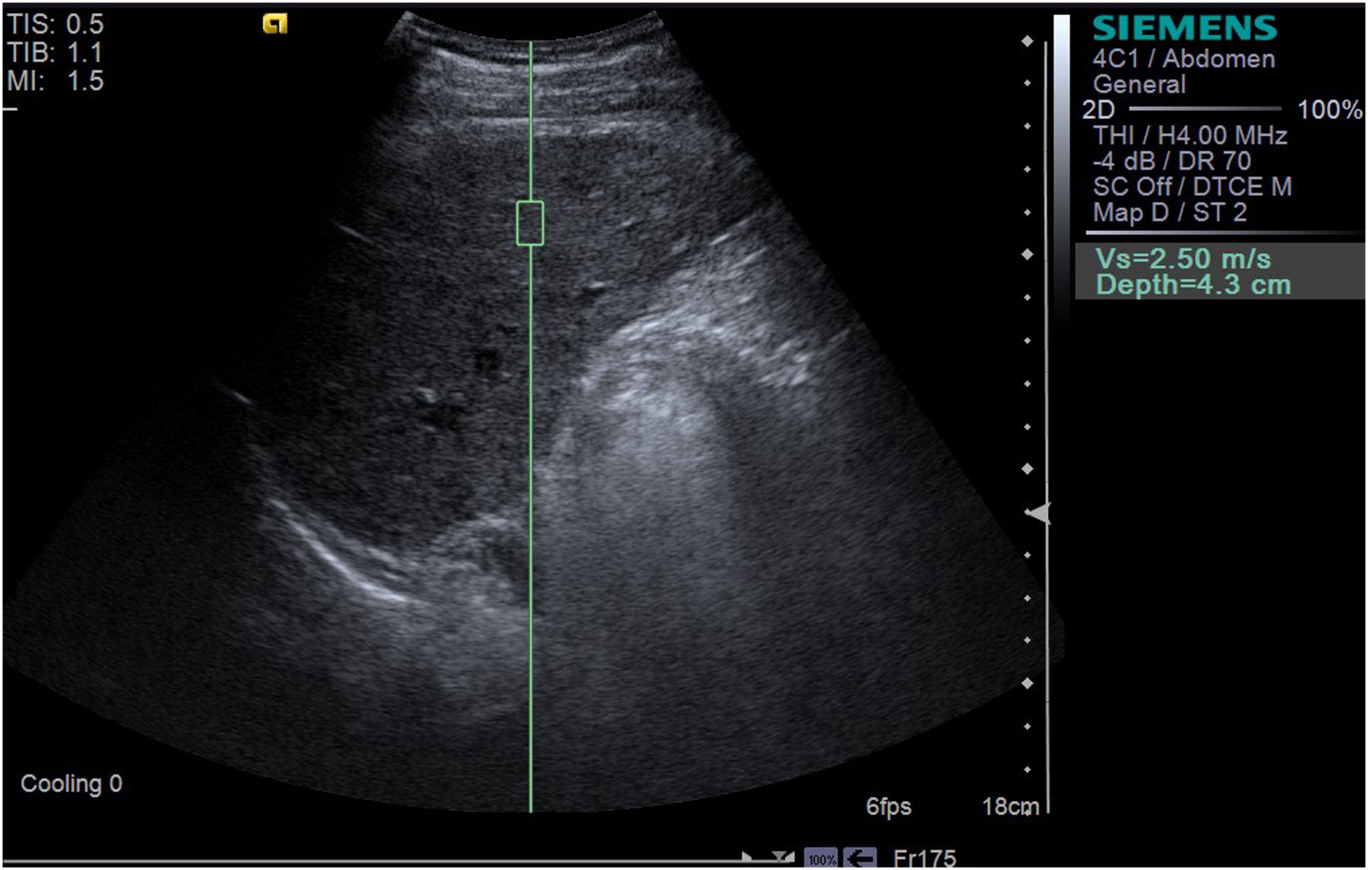
The LSWV of a 35-year-old male patient with one liver nodule, the size of which was 2.4*3.8*3.4cm^3^, caused by HBV. LSWV was 2.50 m/s; ICG-R15 was 43.2% (>10%); and Y-value (multiparametric model) was 28.119 (>16.656).

**Figure 3:**
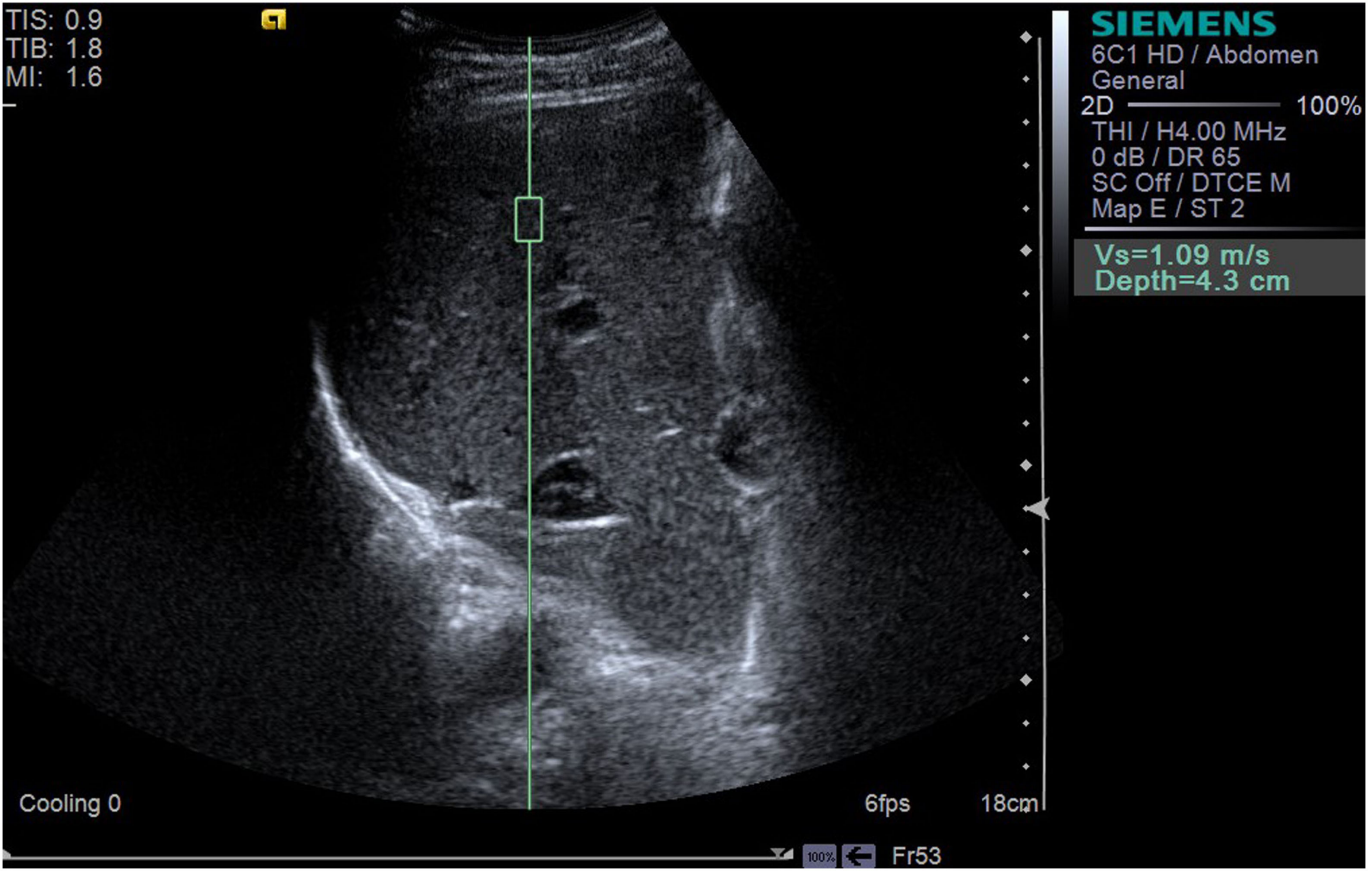
The LSWV of a 61-year-old male shows a LSWV of 1.09 m/s, and ultrasound displays one liver nodule, with a size of 2.8*2.9*3.0 cm^3^. ICG-R15 was 6.0% (<10%); Y-value (multiparametric model) was 4.212 (<16.656).

LSWV was measured using the Siemens Acuson S2000 system (Siemens Healthcare, Erlangen, Germany) equipped with a 1~4 MHz transducer (4C1). During the examination, the patient was placed in the supine position. The region of interest (ROI) was selected in the right lobe and the intrahepatic Glisson system was avoided (17). The ROI was set about 4 cm below the transducer (16). The angle between the sampling line and the liver capsule was adjusted to 90˚. If the tumor lay in the right lobe, the ROI was set to at least 1 cm away from the tumor edge. The patient was asked to hold his breath, and the operator pressed the update button and captured the images of LSWV (**Figures 2**, **3**). Only patients who could hold their breath and tolerate LSWV measurement were enrolled in the study. For each patient, the measurement was repeated 11 times. A ratio of the interquartile range to the median (IQR/M) less than 30% was considered as a successful measurement. The median was recorded as the value of the LSWV examination (16). LSWV examination was performed by two independent radiologists with at least 3 years elastography experience. If the LSWV examination followed the ICG clearance test, it was at least 24 hours apart.

### 2.3 ICG clearance test

A DDG-3300K Hepatic Functional Reserve Analyzer (Nihon Kohden, Tokyo, Japan) and ICG (25 mg/piece; Dandong Yichuang Pharmaceutical Co., Ltd, Dandong, China) were used for ICG-R15. ICG was dissolved in normal saline to the final concentration of 5 mg/mL. The patient fasted for 8 hours prior to the test and was kept in the supine position during the test. The photosensitive probe was connected to the patient’s cleaned ala nasi, ICG was immediately injected at a dose of 0.5 mg/kg via the median cubital vein, and the ICG-R15 was recorded subsequently. In this study, we chose ICG-R15 as golden standard for hepatic reserve function.

### 2.4 Portal vein diameter

The maximum internal diameter of the portal vein was measured in front of the inferior vena cava in the hilar of the liver at 8 hours of fasting (18).

### 2.5 Biochemical tests

Biochemical tests were obtained to evaluate liver function, including alanine aminotransferase (ALT), aspartate transaminase (AST), alkaline phosphatase (ALP),

γ-glutamyl transpeptidase (γ-GGT), albumin (ALB), and prothrombin time (PT).

### 2.6 Tumor volume

The tumor nodule was assumed to be an ellipsoid. Three diameters of the nodule (anteroposterior, transverse, and axial) were measured. Ellipsoid volume was calculated as follows: V= 4π*a*b*c/3, where a, b, and c were the half-length of each diameter. The volume of multiple tumors was the sum of all the nodules.

### 2.7 Statistical analysis

Statistical analysis was performed using SPSS 23.0 (IBM, Armonk, NY, USA) and MedCalc 15.2.2 (MedCalc Software Inc., Ostend, Belgium) software. Normally distributed data were expressed as mean ± standard deviation ( $\bar{x}$ ± s), and abnormally distributed data were expressed as median (interquartile range (IQR)). Data were analyzed using the *t*-test (normal distribution) or the rank-sum test (abnormal distribution). The relationship between multiple clinical factors and hepatic functional reserve were analyzed using multivariate linear stepwise regression, and a multiparametric model was constructed. To compare the diagnostic performance and calculate the diagnostic cutoff values, receiver operating characteristic (ROC) curve analysis was performed. Consistency tests were used to evaluate the predictive effectiveness of the multiparametric model. *P*< 0.05 indicated significant difference.

## 3 Results

### 3.1 Patients’ characteristics

All patients were diagnosed using pathology or imaging examination. Eighty-one patients were confirmed using biopsy pathology or surgical pathology, and forty-two were diagnosed using CEUS, CECT, or MRI. A total of 123 patients (male (99) vs. female (24)) were divided into the study group (male (74) vs. female (18)) and validation group (male (25) vs. female (6)). In the study group, 84 patients had one tumor nodule, seven patients had two, and one patient had three nodules. In the validation group, 27 patients had one tumor nodule and four patients had two tumor nodules. There was no significant difference in patient characteristics between the study group and validation group (**Table 1**).

**Table 1:** Patients’ characteristics.

	Test group	Validation group	t/x^2^/Z	p
	(n=92)	(n=31)		
Age (years)	59.34 ± 10.00	56.97 ± 9.05	-0.31	0.76
Gender			0.001	0.98
Male	74	25		
Female	18	6		
Tumor nodule	101	35		
BCLC staging				
0	7	2		
A	28	12		
B	55	17		
C	2	0		
ICG R15 (%)	7.80 (5.65,25.45)	7.48 (6.10,25.60)	-0.17	0.87
LSWV (m/s)	1.93 ± 0.63	1.94 ± 0.52	-.012	0.90
D_PV_ (mm)	12.04 ± 1.58	11.81 ± 1.47	0.72	0.48
Tumor volume (cm^3^)	14.99 (9.44,21.57)	12.63 (8.82,17.01)	-1.49	0.14
ALT	35.05 (24.00,50.13)	38.20 (23.00,51.20)	-0.26	0.78
AST	38.00 (29.57,51.78)	39.00 (29.70,53.20)	0.00	1.00
ALP	96.15 (77.25,123.75)	95.90 (79.00,115.00)	-0.43	0.66
γ-GGT	57.00 (41.00,86.23)	59.80 (46.20,84.20)	-0.34	0.74
ALB	39.08 ± 6.31	38.57 ± 7.17	0.38	0.71
PT	14.60 (12.80,16.08)	13.60 (11.50,15.40)	-1.29	0.20

LSWV, liver shear wave velocity; D_PV_, hepatic portal vein diameter; ALT, alanine aminotransferase; AST, aspartate transaminase; ALP, alkaline phosphatase; γ-GGT, γ-glutamyl transpeptidase; ALB, albumin; PT, prothrombin time.

### 3.2 Analysis of factors related to hepatic functional reserve and establishment of the multiparametric model

Multivariate linear stepwise regression was used to analyze the clinical parameters. The results showed that LSWV, ALB, and PT were statistically significant in predicting hepatic functional reserve (*P*<0.05). The multivariate regression model was statistically significant (F=45.219, *P*=0.00, adjusted R^2^: 0.593), and the standardized coefficients of LSWV, ALB, and PT were 0.490, -0.199, and 0.313, respectively (**Table 2**). Collinearity analysis showed that the Variance Inflation Factor (VIF) values of LSWV, ALB, and PT were all less than 10, indicating that there was no severe collinearity between the variables. The multiparametric model was formulated as follows: Y= -18.954 + 9.726*LSWV-0.397*ALB+2.063*PT.

**Table 2:** The result of multivariate linear stepwise regression analysis.

	B	Beta	t	p	VIF
LSWV	9.726	0.490	6.723	<0.01	1.190
ALB	-0.397	-0.199	-2.621	<0.05	1.293
PT	2.063	0.313	4.050	<0.01	1.339
F	45.219			<0.01	
Adjusted R^2^	0.593				

LSWV, liver shear wave velocity; ALB, albumin; PT, prothrombin time.

### 3.3 Diagnostic performance of multiparametric model

**Figure 4:**
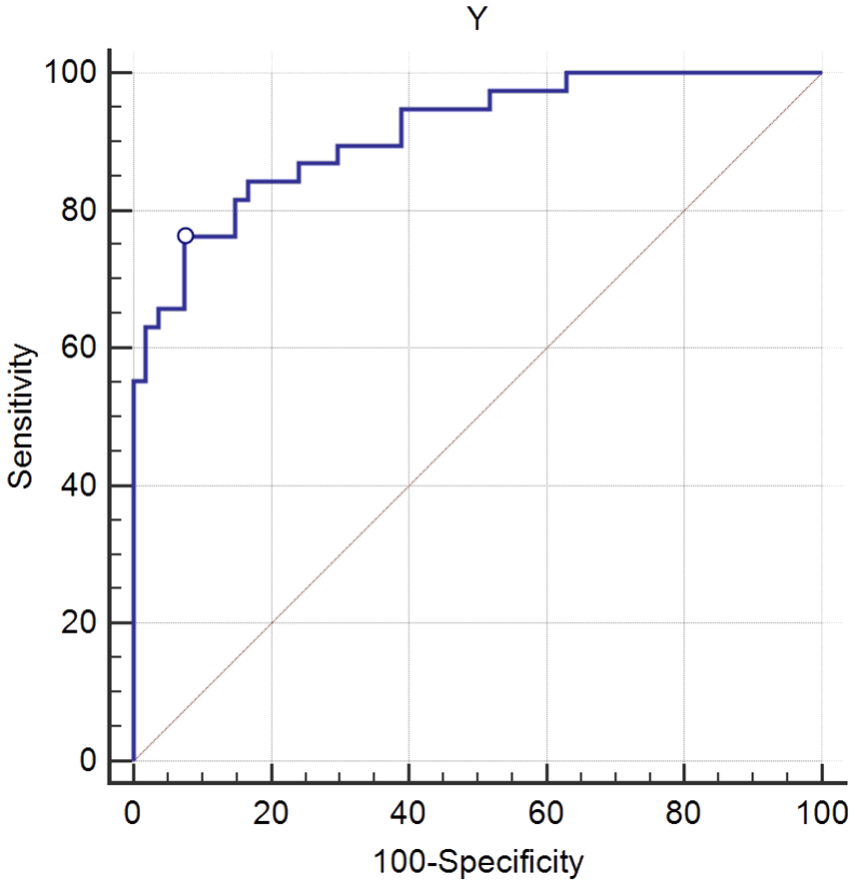
Receiver operating characteristic (ROC) curve analysis of the multiparametric model for the evaluation of hepatic functional reserve. Using 16.656 as the cutoff value for liver functional reserve, the sensitivity and specificity were 0.763 and 0.926, respectively. The value of area under the curve (AUC) was 0.913.

ICG-R15<10% was set as the gold standard, which meant normal hepatic functional reserve. The results of ROC curve analysis showed a high diagnostic performance of the multiparametric model. The values of the AUC for the multiparametric model were 0.913 (95% confidence interval (CI): 0.835-0.962, *P<* 0.01). The cutoff values for the multiparametric model were 16.656 (sensitivity, 0.763; specificity, 0.926) (**Figure 4**).

### 3.4 Consistency test of validation group

In the validation group, ICG R15 less than 10% was used as the gold standard for normal liver functional reserve. Patient’s liver functional reserve was diagnosed as normal or abnormal based on the standard. Then, each patient’s Y value was calculated using the multiparametric model. When the Y value was less than 16.656, it was defined as normal liver functional reserve, and when the Y value was greater than 16.656, it was defined as abnormal. The consistency test was used to analyze the agreement of the two diagnostic methods. The Kappa value was 0.655 (P<0.01), indicating good diagnostic consistency between the two methods.

## 4 Discussion

Hepatic functional reserve is essential for patients undergoing hepatic surgery. A poor hepatic functional reserve is likely to predict postoperative liver failure and even perioperative death. To date, ICG-R15 has been widely used in the clinical evaluation of hepatic functional reserve; however, it has a number of limitations. For instance, it is not very accurate among patients with portal vein thrombosis or with abnormal bilirubin levels. Hepatic ultrasound elastography has been used to evaluate liver fibrosis for about ten years (19–21). Multiple studies have confirmed that LSWV could be utilized to assess hepatic functional reserve (11, 13, 14). The consensus is that there are several clinical parameters related to hepatic function reserve, including LSWV and liver function test (ALT, ALP, ALB, PT, and so on). Unfortunately, previous studies have always focused on LSWV as a single indicator for the assessment of hepatic functional reserve (22). To compensate for the deficiency, in the present study, the results of liver function tests, tumor volume, portal vein diameter, and LSWV were used together to evaluate hepatic functional reserve.

ICG-R15 is the retention rate of indocyanine green in the body at 15 minutes after injection. The smaller the ICG-R15 value, the better the liver reserve function. It is generally believed that when ICG-R15 is less than 10%, the patient can tolerate extensive hepatectomy of four liver segments (7). At present, the ICG clearance test is a commonly used test in clinical practice. Therefore, we chose ICG-R15 as golden standard of hepatic functional reserve in this study. However, ICG-R15 also has its own limitations, as it cannot accurately assess liver functional reserve when portal vein thrombus formation, portal vein cavernous degeneration, and bilirubin elevation occur. This study found that LSWV combined with multiple clinical indicators can better evaluate liver functional reserve, but hyperbilirubinemia can also affect the application of liver shear wave elastography (16). Therefore, for patients with portal vein thrombosis and portal vein cavernous degeneration, we can choose LSWV combined with multiple clinical indicators for liver functional reserve evaluation.

In this study, clinical parameters included LSWV, D_PV_, ALT, AST, ALP, γ-GGT, ALB, PT, and tumor volume. LSWV is used to assess the degree of liver fibrosis and hepatic functional reserve (11, 23); D_PV_ is used to assess the portal hypertension which affects the portal venous blood flow. Portal venous blood flow is related to liver functional reserve; ALT and AST are important indexes for liver cell injury, and increased ALT and AST levels indicate damage to hepatic parenchymal cells; ALP and γ-GGT levels indicate biliary obstruction and cholestasis at any level from the capillary bile duct to the common bile duct; ALB and PT levels reveal the synthetic function of liver (24–26). The above-mentioned parameters are all associated with hepatic functional reserve, and they were accordingly involved in the present study. In addition, the volume of liver tumors was also included as a related factor. As a known fact, the total number of hepatocytes in adult liver tissues is stable. In the case of liver tumors, the hepatocytes surrounding the tumor are squeezed and destroyed. If the tumor size is large enough, then a large number of hepatocytes would be affected and the hepatic functional reserve would be influenced. Therefore, LSWV, portal vein diameter, biochemical indexes, and liver tumor volume were chosen to be used in this study simultaneously.

Multivariate linear stepwise regression was used to analyze the clinical parameters. The results showed that LSWV, ALB, and PT were statistically significant in the evaluation of the hepatic functional reserve (*P*<0.05). The multiparametric model was formulated as follows: Y= -18.954 + 9.726*LSWV-0.397*ALB+2.063*PT. The standardized coefficient of LSWV was 0.490, which was higher than ALB (-0.199) and PT (0.313), indicating that among these factors, the influence of LSWV on hepatic functional reserve was more noticeable than ALB and PT. This is generally consistent with the reports of previous studies, which have demonstrated that LSWV has a high diagnostic performance for hepatic functional reserve (13, 15). The consistency test showed the multiparametric model had a good predictive value and the Kappa value was 0.655.

The study showed that tumor volume was not statistically significant in the evaluation of hepatic functional reserve. This result was different from the presumption. It was presumed that hepatic tumors might influence the hepatic functional reserve. However, the tumor volume apparently didn’t play a significant role in this study. The reason of contradiction might be that the tumor volume was much smaller than liver volume. In the present study, the shape of the tumor nodules were thought to be ellipsoid, the largest diameter of a single tumor was ≤ 5cm, or the sum of the largest diameter of multiple tumors was ≤ 5cm. The maximum tumor volume in this study clearly did not exceed 65cm^3^. The liver volume of healthy Chinese individuals is about (1,050.7 ± 214.3) cm^3^ (27). So, the tumor volume in our study was only<6% of the total liver size. The liver is well known to have strong compensatory capacity. These results indicated that tumor volume (≤ 5 cm) was not statistically significant in the evaluation of hepatic functional reserve. If the liver tumor was large enough to squeeze and destroy a greater number of liver cells, the remaining liver cells might not be sufficient to compensate for liver function. We suspect the hepatic functional reserve would be affected at that point, even if LSWV of the liver parenchyma was normal.

This study investigated hepatic functional reserve using a combination of clinical parameters simultaneously, including LSWV, D_PV_, ALT, AST, ALP, γ-GGT, ALB, PT, and tumor volume. The limitations of the study are as the following: (1) The enrolled patients all had small liver tumors with a tumor size less than 5cm; (2) To avoid inaccurate ICG measurement and LSWV measurement, patients with abnormal bilirubin levels were not included in this study; (3) Patients with liver congestion, acute hepatitis, and infiltrative liver disease were not included; (4) This study only investigated the diagnostic performance of the multiparametric model when ICG-R15 was > 10%. However, ICG-R15>20%, ICG-R15>30%, and ICG-R15>40% appear in clinical practice. (5) The sample size was relatively small, limiting the generalization of our findings.

In conclusion, for patients with Child-Pugh grade A and normal bilirubin, when the maximum diameter of their liver tumor was ≤ 5 cm, LSWV combined with ALB and PT tests could be used to evaluate hepatic functional reserve, which showed a high diagnostic performance. A poor hepatic functional reserve should be noted when the value of Y parameter in the multiparametric model was greater than 16.656, which is important in assisting the clinical diagnosis and management of liver diseases.

## Data availability statement

The original contributions presented in the study are included in the article/supplementary material. Further inquiries can be directed to the corresponding author.

## Ethics statement

The studies involving humans were approved by Medical Ethics Committee of Henan Provincial People’s Hospital. The studies were conducted in accordance with the local legislation and institutional requirements. Written informed consent for participation was not required from the participants or the participants’ legal guardians/next of kin because elastography has become routine in China. All patients in this study had hepatitis B, and each required elastography to evaluate his liver.

## Author contributions

LY: Software, Methodology, Formal Analysis, Conceptualization, Writing – original draft. HZ: Writing – review & editing. XijZ: Writing – review & editing, Investigation, Formal Analysis. XF: Writing – original draft, Investigation, Data curation. XiaZ: Writing – original draft, Software, Formal Analysis. XW: Writing – original draft, Software, Investigation, Data curation. XR: Writing – original draft, Investigation, Data curation. HY: Validation, Resources, Writing – review & editing. JY: Writing – review & editing, Project administration, Funding acquisition.
